# Microvascular invasion is associated with poor prognosis in renal cell carcinoma: a retrospective cohort study and meta-analysis

**DOI:** 10.3389/fonc.2024.1417630

**Published:** 2024-10-11

**Authors:** Jinbin Xu, Yiyuan Tan, Shuntian Gao, Weijen Lee, Yuedian Ye, Gengguo Deng, Zhansen Huang, Xiaoming Li, Jiang Li, Samun Cheong, Jinming Di

**Affiliations:** ^1^ Department of Urology, The Third Affiliated Hospital, Sun Yat-sen University, Guangzhou, Guangdong, China; ^2^ Department of Urology, The First People’s Hospital of Shaoguan, Southern Medical University, Shaoguan, Guangdong, China

**Keywords:** microvascular invasion, renal cell carcinoma, cancer-specific survival, cohort studies, meta-analysis

## Abstract

**Background:**

This retrospective cohort study and meta-analysis aims to explore the association between microvascular invasion (MVI) and clinicopathologiccal features, as well as survival outcomes of patients with renal cell carcinoma (RCC).

**Material and methods:**

The retrospective cohort study included 30 RCC patients with positive MVI and another 75 patients with negative MVI as controls. Clinicopathological features and follow-up data were compiled. The meta-analysis conducted searches on PubMed, Cochrane Library, Web of Science, Embase, and WanFang Data from the beginning to 30 September 2023, for comparative studies relevant to MVI patients. The Newcastle-Ottawa Scale and Egger Test were used to assess the risk of biases and certainty of evidence in the included studies.

**Results:**

The cohort study showed that MVI was associated with advanced primary tumor stage, high pathological grades, high tumor size, high clinical symptoms and lymph node invasion (*P <0.05*). Kaplan-Meier analyses demonstrated MVI was associated with worse CSS rates when compared to MVI negative group (*P <0.05*). However, in the multivariate analysis it was not presented as an independent predictor of cancer survival mortality (*P >*0.05). The meta-analysis part included 11 cohort studies. The results confirmed that patients with MVI positive had worse 12 and 60 mo CSS rates (HR_12mo_ = 0.86, 95%CI 0.80–0.92; HR_60mo_ = 0.63, 95% CI 0.55–0.72; *P* < 0.00001). Moreover, the meta-analysis also confirmed that MVI group was associated with higher rate of advanced tumor stage, pathological grades, tumor size diameter, higher rate of clinical symptoms and lymph node invasion (*P <0.05*).

**Conclusions:**

The presence of MVI in renal cell carcinoma patients is linked to poorer survival outcomes and worse clinicopathological features. In spite of this, it does not seem to be an independent predictor for cancer survival mortality in renal cell carcinoma.

**Systematic review registration:**

https://www.crd.york.ac.uk/prospero/display_record.php?ID=CRD42023470640, identifier CRD42023470640.

## Introduction

Renal cell carcinoma (RCC), the primary malignancy of the kidney, is a heterogeneous and complex disease with numerous pathophysiological variants, providing 2–3% of new cases for all cancers annually (1). Surgical resections involving radical (RN) and partial nephrectomy (PN) still dominate the primary options for localized tumors (2). However, 20-40% of non-metastatic cases will demonstrate local or distant recurrence after nephrectomy (3). Clinicopathological features like pathological stage, tumor size, lymph node (LN) metastasis, tumor necrosis, and patients’ clinical symptoms are combined in several long-term follow-up prognostic models (4). While the well-developed models such as SSIGN (Stage, Size, Grade, and Necrosis) and the UISS (University of California Los Angeles Integrated Staging System) have significantly enhanced our capacity to counsel patients during the long-term follow-up period (5, 6), there is still a need for further refinement in prognostication. These models, while valuable, are not particularly accurate in predicting metastatic kidney cancer, and they fail to provide specific estimates of survival chances. Furthermore, as new biomarkers for prognosis are constantly being discovered, the previous models, which did not take into account these new markers, may be less accurate in some cases.

Microvascular invasion (MVI), which is characterized by the invasion of cancer cells into the walls of blood vessels or the presence of cancerous emboli within the vessel lumen, has been identified as a potential risk factor in RCC. In a 40-month-long follow-up descriptive and analytical study involving 221 patients, Bengió RG (7) concluded that MVI is associated with unfavorable tumor characteristics. Similarly, Eisenberg MS (8) and Kwon SY (9)reported a positive correlation between MVI and cancer-specific survival (CSS) rates in long-term follow-up. Many of these studies have demonstrated a strong prognostic impact of MVI on the progression of RCC. Despite these findings, the discourse surrounding the role of MVI in RCC yet warrants exploration.

In this retrospective cohort study, we aim to explore the potential association between MVI and RCC, specifically focusing on the survival rate and clinicopathological features in patients with MVI. Moreover, we took a further step by conducting a meticulous evaluation of the relationship between MVI and RCC through a systematic review and meta-analysis. By incorporating data from all published observational cohort studies, our analysis aims to provide a more robust and reliable conclusion. This comprehensive approach strengthens the evidence and enhances our understanding of the connection between MVI and RCC.

## Methods

### Retrospective cohort study

#### Study cohort

We retrospectively reviewed all case records from adult patients who were recorded between June 2018 and September 2021 in The Third Affiliated Hospital of Sun Yat-sen University. The inclusion criteria for patients in retrospective studies were as follows: (1) Patients with renal cell carcinoma underwent partial or radical nephrectomy surgery. (2) No targeted therapy chemotherapy or radiotherapy was given before surgery.

#### Clinical data and statistical analysis

Detailed patient history was extracted by two researchers from electronic records in hospital database. These included: sex, age, smoking history, as well as tumor-related indicators (such as maximum tumor diameter, microvascular invasion, peripheral fat infiltration, and tumor stage), since the aforementioned factors, especially the tumor-related features, are closely related to survival time. Preoperative imaging, including computerized axial tomography was also needed. All pathological results were centrally reviewed and reconfirmed by another one pathologist. The patients were then categorized into positive and negative groups based on the presence or absence of MVI, and CSS rate was defined as the time to the date the patient died of disease.

Statistical analysis was conducted using SPSS Statistics 27.0. A significance level of *P* < 0.05 was considered for all aforementioned outcomes. The analysis of variable categories was executed either by Chi-square method or Fischer test when most suitable. Student’s t-test was used for comparison of continuous variables. The Kaplan-Meier method was employed to assess cancer-specific survival, and the Log-rank test was used to compare the differences between groups. To predict risk factors of metastases and cancer-specific mortality, after univariate analysis, multivariate analysis was carried out using the Cox proportional hazards method.

### Systematic review and meta-analysis

#### Search strategy

The systematic review part was registered in the PROSPERO prospective register of systematic reviews (CRD42023470640), conducted based on a predefined protocol and performed following PRISMA guidelines (10). We searched PubMed, Embase, Web of Science, Cochrane Library and WanFang Data from inception to 30 September 2023. “Carcinoma, renal cell,” “Microvascular invasion,” and “Nephrectomy,” were used as the keywords for the search. Additionally, a manual search of references from identified clinical trials and included studies was performed to identify further potentially relevant literature.

#### Inclusion criteria and exclusion criteria

The inclusion criteria were as follows: (1) Participants (P): patients with renal carcinoma and the minimum number of cases were more than 40 per study. (2) Interventions and comparisons (I and C): MVI exposure versus MVI absent. (3) Outcomes (O): CSS rate, disease-free survival (DFS) rate, etc. (4) Study design (S): Population-based prospective or retrospective cohort study were eligible for inclusion if they had at least 1 year of follow-up and examined the relation of MVI exposure with patient’s survival rate. Exclusion criteria: (1) Available patient-related data (accurate survival rate and detailed patient baseline characters) could not be extracted. (2) When the same cohort is present at multiple publications, only the longest follow-up or analysis covering the largest number of participants is involved.

#### Data extraction and quality assessment

Two reviewers independently conducted the abstract screening, full-text evaluation, data extraction, and cross-checking using the inclusion criteria with Noteexpress software. Any disagreements regarding the eligibility of an article were discussed, and a final consensus was reached with a third author. The following details were recorded: study design, sample size, duration of follow-up, characteristics of the study population (age, sex, tumor diameter, level of pathological grade, primary tumor classification, LN metastasis rate, peripheral fat invasion rate and clinical symptom rate, etc.) and intervention technique (RN or PN). The Newcastle-Ottawa Scale (NOS) was used to estimate the risk of bias and level of evidence of included studies. Studies scoring between 1-3 points are considered low-quality research, between 4-6 points are deemed moderate-quality research, and a maximum score of 9 points on the NOS reflects the highest study quality.

#### Data synthesis and analysis

Statistical analysis was conducted using Review Manager 5.0 and STATA 12.0 software. Summary measures were presented as relative risks (RRs) with 95% confidence intervals (CIs). A *P*-value less than 0.05 was considered statistically significant. Statistical heterogeneity between studies was calculated using the Chi-square test, with significance set at a *P*-value less than 0.10. Heterogeneity was quantified using the *I* ^2^ calculation. If *I*²of a particular indicator is less than 50%, the results of this indicator can be deemed as relatively stable, where *I* ^2^ greater than 50% indicates significant heterogeneity. Then A one-by-one study exclusion sensitivity analysis was performed to analyze heterogeneity. We excluded each included study one by one, noting the alterations, and then recalculated the combined effect size of the remaining studies to observe any changes. Subgroup analysis based on whether the RCC had metastasized or not was also performed in specified characters to compared the survival rate between two groups. Finally, the Egger test were used to evaluate publication bias, with a *P*-value less than 0.1 defined as significant publication bias.

## Results

### Retrospective cohort study

#### The basic character of the cohort study

Ultimately, our cohort study encompassed 105 patients, comprising 67 males and 38 females, with an average follow-up duration of 30 months. The mean age was 57 years old (range 28–88). There were 30 (28.6%) patients with MVI, while 75 (71.4%) patients did not present with MVI. At the time of analysis (September 2023), 21(20.0%) patients had died from RCC. Detailed baseline data of the entire cohort is given in **Table 1**.

**Table 1:** Association of different variables and microvascular invasion.

Variable	MVI(+) (n= 30)	MVI(-) (n= 75)	P
Age (mean)	58.56 ± 11.76	55.82 ± 12.52	0.31
Sex			0.95
Male	19 (63.3%)	48 (64.0%)	
Female	11 (36.7%)	27 (36.0%)	
Clinical manifestations			<0.001
Symptomatic	24 (80.0%)	24 (32.0%)	
Incidental	6 (20.0%)	51 (68.0%)	
Surgery type			<0.001
Radical nephrectomy	27 (90.0%)	27 (36.0%)	
Partial nephrectomy	3 (10.0%)	48 (64.0%)	
Tumor diameter (cm)	8.41 ± 2.98	4.82 ± 2.55	<0.001
>7 cm	20 (66.7%)	10 (13.3%)	
T-stage			<0.001
T1	9 (30.0%)	63 (84.0%)	
T2	4 (13.3%)	10 (13.2%)	
T3	14 (46.7%)	1 (1.4%)	
T4	3 (10.0%)	1 (1.4%)	
N-stage			
pN0/pNX (cN0)	21 (70.0%)	65 (86.7%)	0.04
N1	9 (30.0%)	10 (13.3%)	
M-stage			
M0	27 (90.0%)	74 (98.7%)	0.126
M1	3 (10.0%)	1 (1.3%)	
WHO/ISUP level			
I-II	9 (30.0%)	64 (85.3%)	<0.001
III–IV	21 (70.0%)	11 (14.7%)	
Positive peripheral fat infiltration	13 (43.3%)	4 (5.3%)	<0.001
History of smoking	11 (36.7%)	20 (26.7%)	
BMI	23.31 ± 3.19	24.22 ± 2.84	0.15

WHO/ISUP level, World Health Organization/International Society of Urological Pathology classification.

#### Comparison of clinical and pathological features in MVI positive vs MVI negative patients

The predominant histological subtype in this study was clear-cell carcinoma, with 99 patients (94.3%). Most MVI group patients were proposed to seek hospital help because of insufferable clinical symptoms like backache (43.3%), abdominal pain (6.7%), or hematuria (30.0%, **Table 1**). While in the counterpart group, more RCC patients were exposed because of annual health checks (68.0%, *P <*0.001, **Table 1**). At the final analysis, among patients with MVI, postoperative pathological examination revealed that a total of 9 patients (30%) had concurrent peritumoral LN invasion, while in the MVI absent group, only 10 patients (13.3%) exhibited (**Table 1**). Similarly, regarding peripheral fat infiltration, a higher proportion was observed in the MVI positive group, with only 5.3% in the MVI negative group (*P <*0.05). In addition, our study found that the average tumor diameter in the MVI positive group was larger (*P <*0.001). And a higher rate of T and N stage (*P <*0.05) were also observed in patients with MVI (**Table 1**).

#### Association of MVI with survival outcome

**Figure 1:**
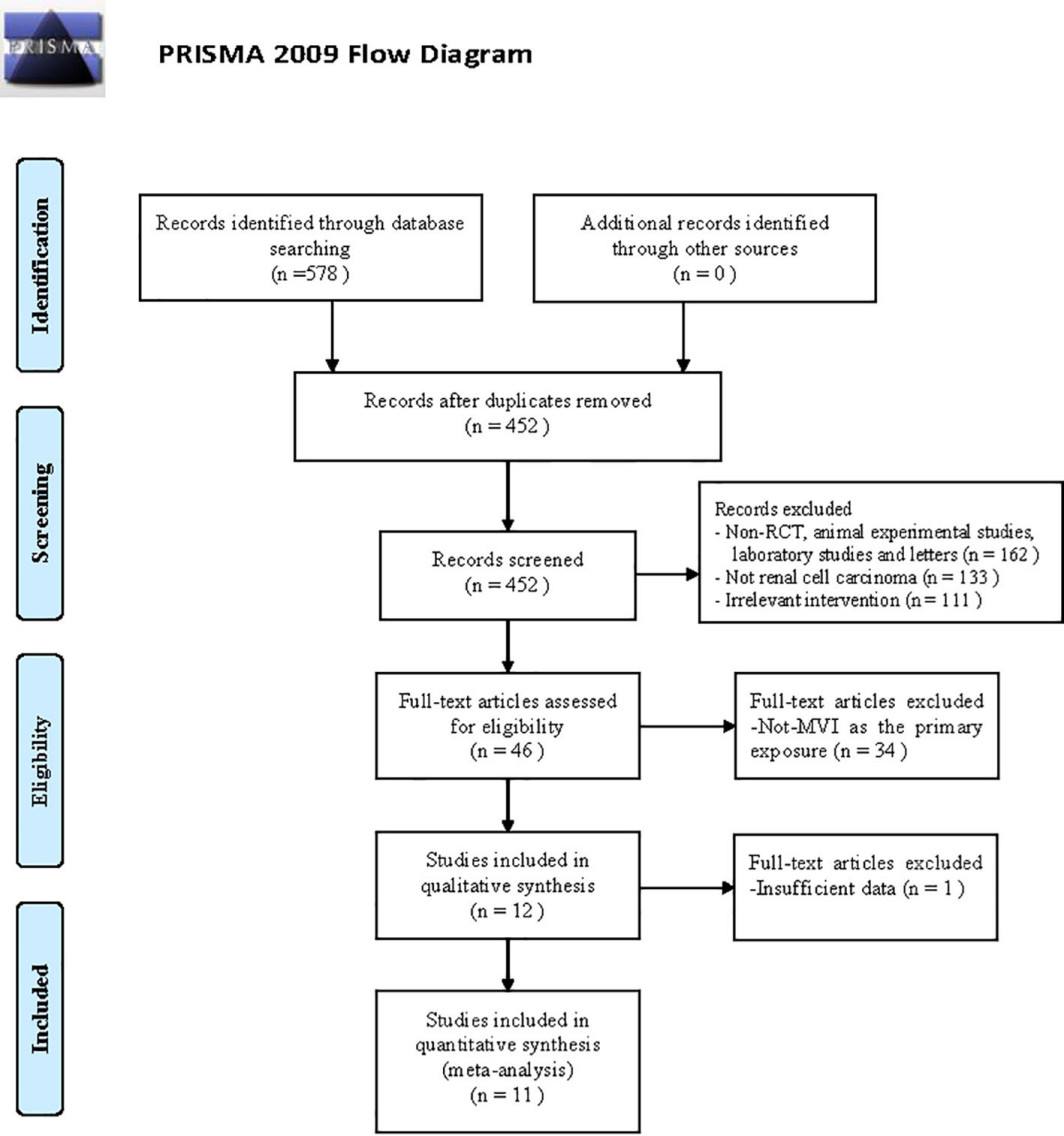
Cancer-specific survival in patients with MVI positive vs MVI negative.

Kaplan-Meier analyses demonstrated worse CSS rate for patients with MVI at 12 mo when compared to the MVI negative group (90.0% vs 100.0%). The 24-month CSS rate in the MVI positive group and MVI negative group were also statistically significant (log-Rank test *P <*0.005) (**Figure 1**). The multivariate analysis utilizing the Cox proportional method to identify risk factors for cancer-specific mortality in RCC patients revealed that a tumor diameter greater than 7 cm (HR = 0.210, 95% CI: 0.053-0.7999, *P <*0.05), advanced WHO/ISUP level (HR = 6.332, 95% CI: 1.319-30.407, *P <*0.05) and perirenal fat infiltration (HR = 4.922, 95% CI: 1.214-19.947, *P <*0.05) were the independent predictors for death related to renal cancer according to **Table 2**. However, our findings did not provide evidence that MVI was an independent predictor for cancer survival mortality (HR = 0.574, 95% CI: 0.140-2.343, *P >*0.05, **Table 2**). Additionally, factor such as the presence of clinical symptoms did not emerge as independent predictors for cancer-specific mortality either.

**Table 2:** Multivariable Cox proportional hazard regression model in all patients.

Variable	HR	Lower 95% CI	Upper 95% CI	*P*-value
Maximum diameter (>7 cm)	0.210	0.057	0.799	0.024
Clinical symptomatic	0.547	0.158	1.895	0.342
Microvascular invasion	0.574	0.140	2.343	0.439
Body mass index	0.901	0.739	1.100	0.306
Perirenal fat infiltration	4.922	1.214	19.947	0.026
Smoking history	0.774	0.291	2.061	0.608
T-stage	1.358	0.336	5.491	0.667
WHO/ISUP level	6.332	1.319	30.407	0.021
N-stage	2.373	0.382	14.739	0.354

### Systematic review and meta-analysis

#### Baseline of included studies

**Figure 2:**
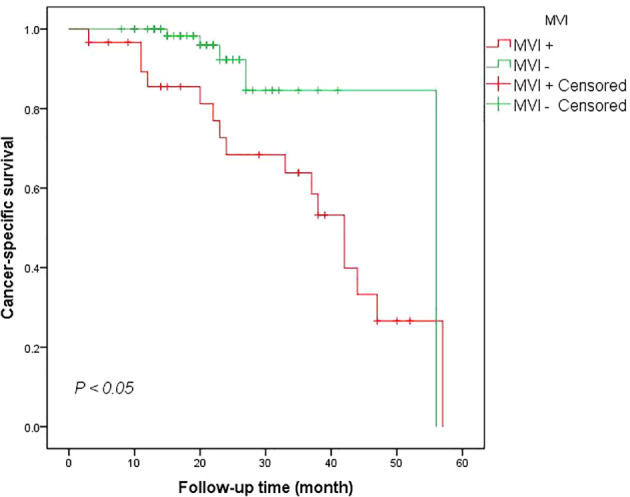
Study selection flowchart.

**Figure 2** presented a flow diagram that outlines the systematic review process. Initially, a total of 578 studies related to MVI in RCC were identified through the search. After screening the titles, abstracts, and examining the full text. A total of 11 articles (3, 7, 8, 11–18) were included in the review, involving 7013 participants. The baseline characteristics of the comparative studies are presented in **Supplementary Table 1**. The average duration of follow-up varied from 18 to approximately 153 months. The majority of patients included in the studies had clear-cell renal carcinoma histological subtype (ccRCC), and most tumors were classified as pathological grade II or III. At final diagnosis, 24.8% (1793/7013) of patients were confirmed to have MVI. Six studies reported the number of patients with tumors larger than 7 cm in diameter.

#### Analysis of clinicopathological features and follow-up results

**Figure 3:**
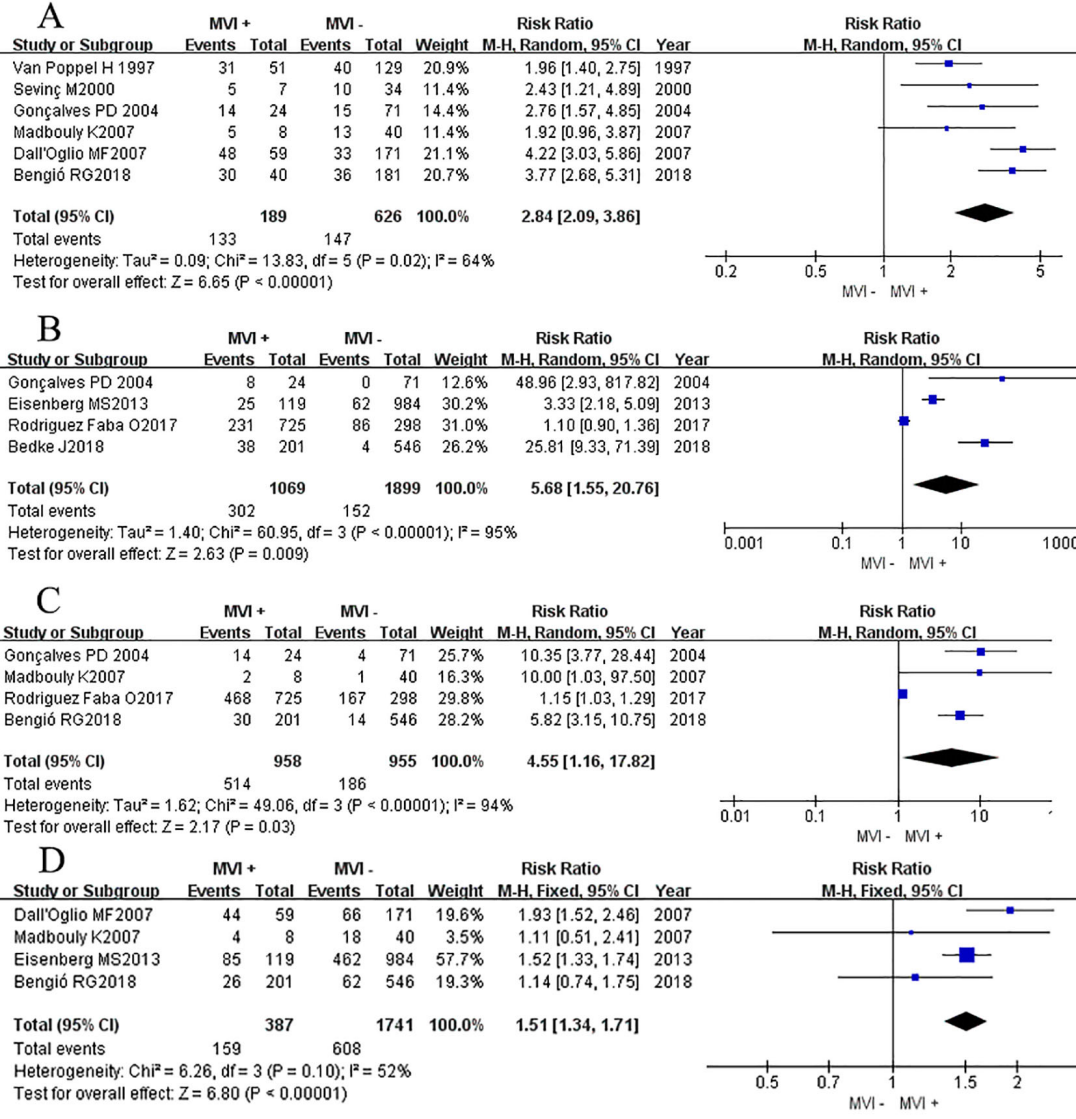
Forest plot of outcomes between the MVI positive and MVI negative. **(A)** 12 mo cancer-specific survival rate, **(B)** 60 mo cancer-specific survival rate, **(C)** 12 mo cancer-specific survival rate of non-metastatic RCC group, and **(D)** 60 mo cancer-specific survival rate of non-metastatic RCC group.

Seven studies (3, 7, 11–13, 15, 16) compared the CSS rate at 12 months between groups with and without MVI. The results showed a significant heterogeneity between the different groups (*P* = 0.004, *I* ^2^ = 69%). Sensitivity analyses were conducted, but no obvious heterogeneity was found in any particular study. The overall effect showed that groups with MVI had a lower survival rate compared to groups without MVI (RR_RE_= 0.86, 95%CI 0.80–0.92, *P <*0.0001 **Figure 3A**). Eight studies (3, 7, 8, 11–13, 15, 16) provided data on the association between MVI and the CSS rate at 60 months. A significant decrease in the CSS rate among patients with MVI was observed compared to those with MVI-negative tumors (RR_RE_ = 0.63, 95% CI 0.55–0.72; *P <*0.00001, **Figure 3B**). A Chi-square test indicated significant heterogeneity between the two groups (*P* = 0.0008, *I* ^2^ = 72%). We thoroughly examined each included study and found no potential literature that could explain this heterogeneity. Additionally, we performed subgroup analysis based on whether the RCC had metastasized or not. In the sub-group of non-metastatic RCC, we found that the MVI group also suffered a lower 12 mo and 60 mo CSS rates (*P_12mo <_* 0.0001, *P*_60mo <_0.05; **Figures 3C, D**).

**Figure 4:**
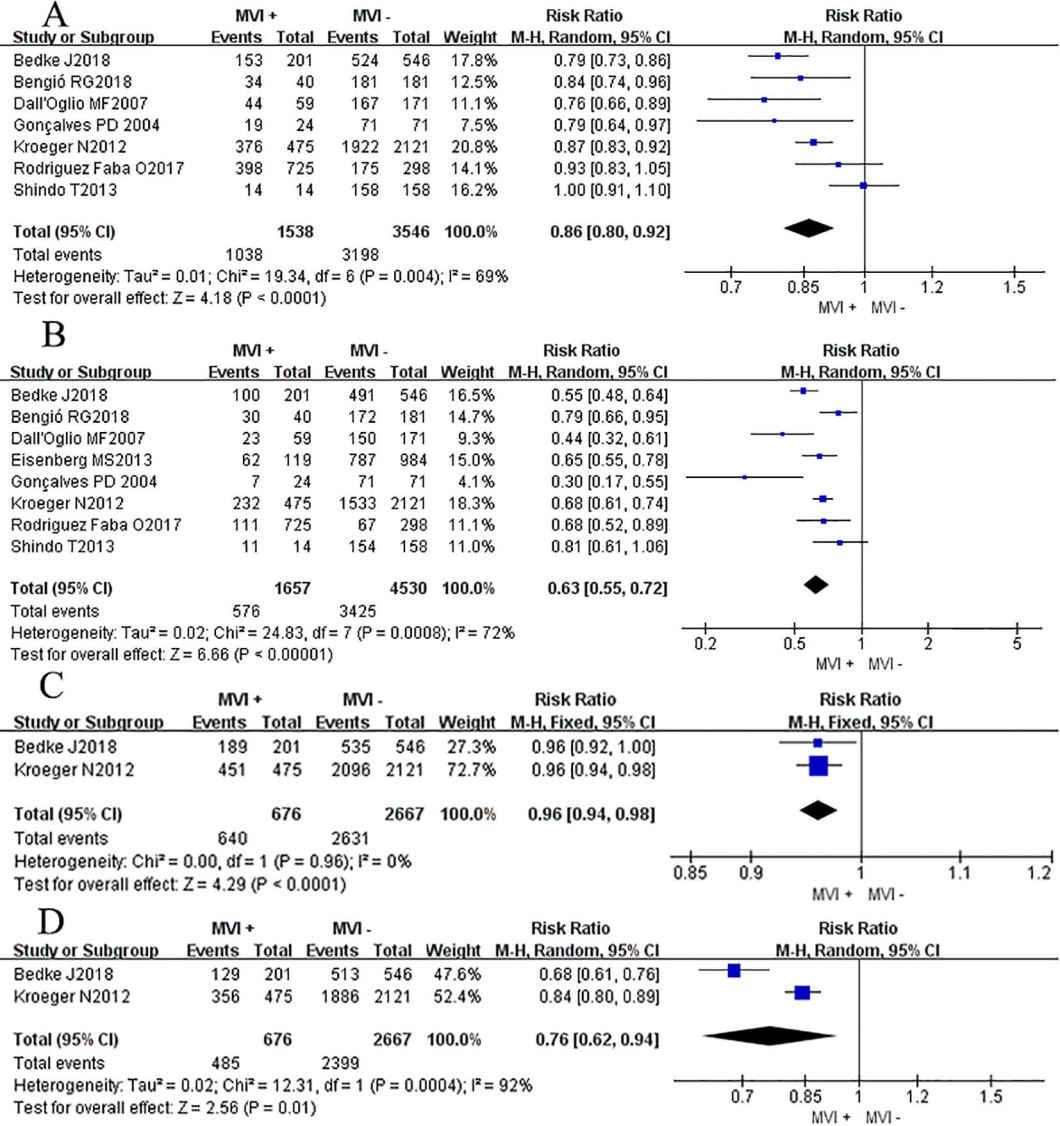
Forest plot of outcomes between the MVI positive and MVI negative. **(A)** 12 mo disease-free survival rate, and **(B)** 60 mo disease-free survival rate, **(C)** the rate of advanced pathological grade, **(D)** the rate of advanced tumor classification.

Three studies (14, 15, 18) reported the DFS rate at 12 months and 60 months, we found that there was no significant difference between two groups at 12 months DFS rate (RR_RE_ = 0.87, 95% CI 0.75-1.02; *P* = 0.09, **Figure 4A**), but after a longer period of follow-up time, at 60 months, results showed that the MVI positive group had a lower DFS rate at 60 months compared to their counterparts (RR_RE_= 0.45, 95% CI 0.29–0.70; *P* = 0.0003, **Figure 4B**). Chi-square test analyses showed a significant difference in heterogeneity between different studies (*P_12mo_* = 0.03, *I* ^2^ = 71%; *P_60mo_* = 0.06, *I* ^2^ = 65%). After eliminating relevant literature one by one, no potential sources of heterogeneity were identified, and a random effects model was used for analysis.

Nine studies (3, 7, 8, 11, 14–18) reported the association between the high level of pathological grade (III-IV) and MVI, and found that patients with positive MVI tumors were associated with a higher rate of high pathological grade (III-IV) level compared to those without MVI (RR_RE_ = 2.53, 95% CI 1.75-3.67; *P <*0.00001, **Figure 4C**). Chi-square test analyses showed a significant difference in heterogeneity between different studies. No potential literature was found that could explain the observed heterogeneity, so a random effects model was used for analysis finally.

Eight studies (3, 7, 8, 11, 14, 15, 17, 18) provided data on the association between the advanced primary tumor classification (T3-T4) and MVI. Interestingly, patients with positive MVI tumors were presented a comparable rate of high primary tumor classification (T3-T4) compared to their counterparts (RR_RE_ = 3.21, 95% CI 0.62-16.74; *P <*0.17, **Figure 4D**). While Chi-square test analyses showed a significant difference in heterogeneity between different studies (*P <*0.00001).

**Figure 5:**
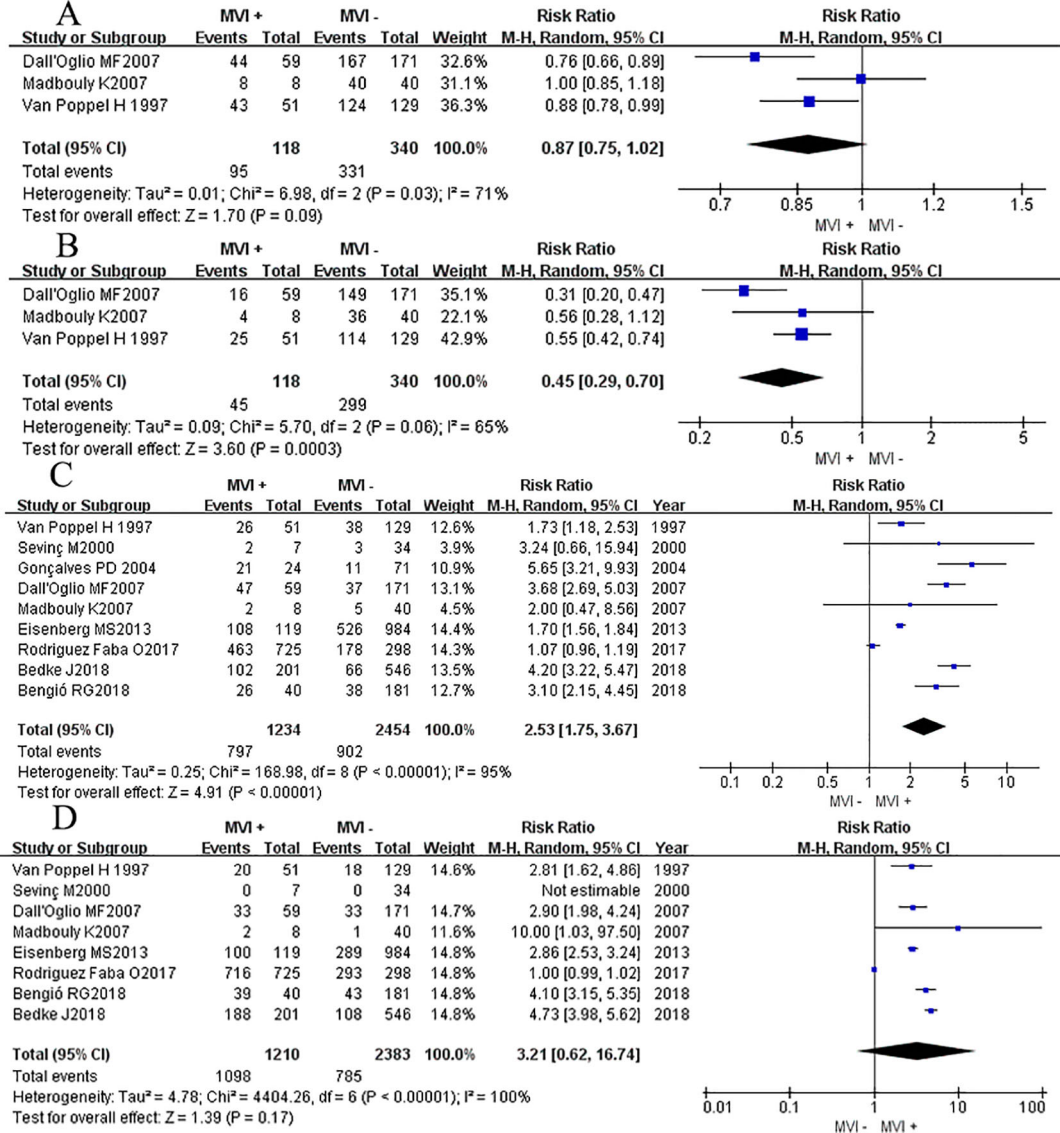
Forest plot of outcomes between the MVI positive and MVI negative. **(A)** the rate of large tumor diameter, **(B)** the rate of lymph node metastasis, **(C)** the rate of peripheral fat invasion, and **(D)** the rate of clinical symptom.

Six studies (7, 14–18) reported the association between the max tumor diameter (>7cm) and MVI. Outcomes showed that patients with positive MVI tumors were associated with a higher rate of max tumor diameter compared to those without MVI (RR_RE_ = 2.84, 95% CI 2.09-3.86; *P <*0.00001, **Figure 5A**). Similarly, Chi-square test analyses showed a significant difference in heterogeneity between different studies (*P <*0.00001).

Four studies (3, 8, 11, 16) reported the association between the LN metastasis rate and MVI. Outcomes showed that patients with positive MVI tumors were associated with a higher rate of LN metastasis compared to those without MVI (RR_RE_ = 5.68, 95% CI 1.55-20.76; *P* = 0.009, **Figure 5B**). Significant heterogeneity differences were found between different studies (*P <*0.00001, *I* ^2^ = 95%), a random effects model was used for analysis.

Four studies (7, 11, 14, 16) reported the association between the peripheral fat invasion rate and MVI, and found that patients with positive MVI tumors were associated with a higher rate of perirenal fat invasion rate compared to those without MVI (RR_RE_ 4.55, 95% CI 1.16-17.82; *P* = 0.03, **Figure 5C**). Significant heterogeneity differences were found between different studies (*P <*0.00001, *I* ^2^ = 95%), a random effects model was used for analysis.

Four studies (7, 8, 14, 15) reported the association between the clinical symptom rate and MVI in patients with RCC and found that patients with positive MVI tumors were associated with a higher rate of clinical symptom happening compared to those without MVI (RR_RE_ = 1.51, 95% CI 1.34-1.71; *P <*0.00001, **Figure 5D**). Chi-square test analyses showed no significant difference in heterogeneity between different studies (*P* = 0.10, *I* ^2^ = 52%), a fixed effects model was used for analysis.

The Egger test was utilized to evaluate publication bias, and the findings indicated no indications of publication bias among the results of the 11 studies included in this meta-analysis.

## Discussion

This retrospective cohort study and meta-analysis aimed to investigate the association between MVI, survival rate, and other clinicopathological features in patients with RCC, especially to provide comprehensive evidence on the impact of MVI on long-term oncological outcomes. The result revealed a consistent association between MVI and unfavorable long-term survival outcomes. Patients with MVI-positive RCC suffered significantly lower survival rates compared to those without MVI. However, our research also showed that, there was no direct evidence to prove that MVI is an independent predictor for cancer survival mortality. This suggested that we need to be more cautious and comprehensive, fully considering the combined effects of various factors, including pathological characteristics and tumor stages when applying this indicator. By segregating patients into MVI-positive and MVI-negative groups, we are able to conduct postoperative risk stratification in a targeted manner. Additionally, further follow-ups can be initiated to pinpoint the recurrence timing in a more expeditious fashion.

It has been proven that advanced TNM stage, larger size of the primary tumor, advanced nuclear grade, and the presence of sarcomatoid elements are correlated with the worse prognosis of renal cancer (19). MVI, as it always indicates a more aggressive tumor behavior and a higher risk of metastasis phenomenon, although it has been widely recognized as an important prognostic factor in various urological and non-urological malignancies, its role in RCC has remained a subject of discussion (20). Our findings revealed that patients with MVI present had significantly worse survival outcomes. Meta-analysis confirmed the outcomes we were interested in, like 12-month and 60-month CSS rates, 12-month and 60-month DFS rates (*P<0.05*), etc. These results aligned with a recent study (21), which demonstrated a strong negative correlation between MVI and tumor characteristics in non-metastatic RCC. However, upon conducting a thorough multivariate analysis, we observed that the factor did not emerge as a significant independent predictor of cancer-specific mortality (HR = 0.574; P= 0.439). This finding is in line with current research finding (7). Yet there are also contradictory studies. Rodriguez Faba O (11) found that MVI was associated with worse cancer-specific mortality. With a 57.4% 5-year CSS rate and the overall survival (OS) rate is 48.4%. Similar results were observed in the studies conducted by Kroeger N (13) and Sorbellini M (22). They pointed out that MVI is associated with a poorer prognosis and is calculated to be an independent predictor of metastatic spread with a higher risk of recurrence and metastasis. Several factors may contribute to the contrary results observed. The adjustment for confounders is one such factor. Kroeger N’s study found that the statistical significance of MVI as an independent predictor of CSS diminished after adjusting for multiple variables, suggesting that other factors might explain the observed association. Conversely, the other research showed MVI to remain an independent predictor even after adjustment, indicating a more robust effect. Rodriguez Faba O’s inclusion of macrovascular invasion in their analysis potentially influenced the results, as the impact of both macrovascular invasion and MVI on survival outcomes was significant only in univariate analysis, not in multivariate analysis, suggesting overlapping effects. Additionally, the duration of follow-up and the sample size of studies can affect conclusions, with longer follow-up periods and larger sample sizes potentially better equipped to detect subtle differences in survival outcomes.

Our findings have found that patients displaying positivity for MVI bear a heightened susceptibility to LN metastasis (*P< 0.05*). This discovery is indeed noteworthy, for it uncovers a conspicuous concurrence wherein a positive correlation between the two phenomena is discernible. LN metastasis is intricately linked with the distant dissemination of RCC and portends an unfavorable prognosis. Existing evidence substantiates the 5-year survival rate in RCC patients with LN metastasis to range from a meager 5% to a modest 38% (23). Furthermore, several investigations have corroborated that patients afflicted with RCC and accompanying LN metastasis are predisposed to the emergence of distant metastasis (24). In essence, patients afflicted with MVI are at an elevated peril for LN metastasis. The discernment of this link empowers clinicians to stratify patients based on their risks, thereby facilitating diverse follow-up protocols in time and informing proactive implementation of tailored interventions like lymph node dissection (LND) and postoperative targeted chemotherapy (1, 25, 26).

It has been demonstrated that a higher grade of pathological level in ccRCC, according to the WHO/ISUP classification, is indicative of heightened biological aggressiveness and is associated with a worse prognosis (27, 28). RCC tumors were typical divided into two different groups including low-grade (Grade I and II) and high-grade (Grade III and IV) groups. Notably, the high-grade group exhibited a higher risk of recurrence following partial nephrectomy. Our findings indicated that patients classified into the high-grade group have a heightened likelihood of MVI following. Consequently, clinicians must prioritize prompt postoperative intervention, as MVI with Grades III and IV portends an unfavorable clinical prognosis. Tumor size was another important clinical and pathological feature for patients with RCC for many decades (29). Previous studies have demonstrated that primary tumor size was an independent predictor of survival. Small primary tumors, by their nature, tend to possess a heightened level of homogeneity and manifest diminished genomic instability, attributed to a less belligerent progression of the disease. Available evidence confirmed that a 1-cm difference in primary tumor size can lead to a 10–31% relative difference in the risk of death (30). According to the findings of this research, it has been noticed that patients with MVI exhibit a propensity towards possessing tumors of larger sizes. Due to higher levels of clinical symptoms or larger tumor diameter, these patients with MVI are more easily determined by imaging to be invading beyond the kidney and are more likely to be taken for radical resection. At the same time, the clinical TNM staging in these patients was higher than that of the control group, with more patients classified in stages III and IV (60.0% VS 2.7%), which may explain the worse survival rates observed in patients with MVI. In recent years, with the rapid advancement of precision medicine, emerging molecular markers have garnered increasing attention, MUC1, known for its role in cell proliferation and metabolic reprogramming, could interact with or influence the pathological pathways associated with MVI (31). The integration of molecular markers with traditional clinicopathological features, such as tumor size, stage, and grade, may facilitate personalized treatment strategies for RCC patients. Complement system, like pro-angiogenic factors (C3a and C5a), are also other critical players, which may interact with or exacerbate pathways related to MVI (32). Previous research found that both clear cell and papillary RCC upregulate most complement genes relative to normal kidney tissue, suggesting a potential intersection between complement system dysregulation and MVI in influencing tumor behavior and patient outcomes. Targeting the complement pathway could be a novel therapeutic strategy for managing RCC, particularly in cases exhibiting significant MVI.

In summary, we contend that all the aforementioned factors including primary tumor size, metastatic burden, microvascular invasion, perirenal fat infiltration and LN metastasis collectively signify the intrinsic characteristics of the tumor’s biological composition, potentially serving as the key determinant of disease aggressiveness within this particular context. And the emerging molecular markers such as MUC1 and pro-angiogenic factors in the complement system (C3a and C5a) also deserve more attention. Yet we still have to realize that it remains unknown which of these factors serves as the primary driver. Whether one or the other plays a major role, or the two or three promote each other, requires a series of follow-up studies.

This study has several limitations. Firstly, selection bias may impact the generalization of the study’s findings. The retrospective cohort study, which solely considers patients recorded in a single hospital over a specific time period, might have resulted in the exclusion of other crucial populations, thereby introducing additional biases related to regional practices, patient demographics, and access to healthcare resources. These factors could restrict the applicability of the study’s findings to other geographical regions or healthcare systems. Secondly, although the meta-analysis component of the study offers additional statistical strength, it was hindered by heterogeneity among the included studies. The sensitivity analysis failed to effectively discern the studies contributing to the apparent heterogeneity, ultimately leading to a reduced overall credibility. Thirdly, despite adjusting for known confounding variables, the likelihood of residual confounding persists. Including patient comorbidities, treatment regimens, and tumor genetics, may have influenced the observed associations between MVI and RCC outcomes. Hence, the observed effects of MVI on RCC prognosis may be partially attributed to these unmeasured factors. Lastly, while the study focuses on the epidemiological link between MVI and RCC, it offers limited insights into the underlying biological mechanisms connecting these two factors. A profound comprehension of the molecular pathways and cellular interactions involved in MVI and RCC progression is essential for developing more targeted therapies and prognostic biomarkers.

## Conclusion

Based on our findings, it is clear that the presence of MVI in RCC patients is associated with worse survival outcomes and clinicopathological features. However, our research also shows that, based on current evidence, there is no direct information to prove that MVI is an independent predictor for cancer survival mortality. It emphasizes the need for a more cautious and comprehensive approach in understanding and applying this indicator. Further research is needed to fully understand the underlying mechanisms and to determine the most effective treatment strategies for patients with MVI-positive RCC.

## Data Availability

The original contributions presented in the study are included in the article/**Supplementary Material**. Further inquiries can be directed to the corresponding author.
