# Supplementary material for: Microvascular invasion is associated with poor prognosis in renal cell carcinoma: a retrospective cohort study and meta-analysis
Source: Front Oncol. 2024 Oct 11;14:1417630. doi: 10.3389/fonc.2024.1417630 (PMC11502461; doi:10.3389/fonc.2024.1417630)
Supplement: Supplementary file 1 [file Table1.docx]

**Supplementary Table 1** The basic character of included studies.

| Study | Country | Patients | Age | T Stage (T1-2/T3-4) | Histological Grade(III-IV) | Histological Subtype(ccRCC) | Tumor Diameter (0-7cm/＞7cm) | NOS Point |
| --- | --- | --- | --- | --- | --- | --- | --- | --- |
|  |  | T/C | T/C | T/C | T/C | T/C | T/C |  |
| Van Poppel H | Turkey | 51/129 | 60 | 31/20 | 26 | -- | 20/31 | 7 |
|  |  |  |  | 111/18 | 38 | -- | 89/40 |  |
| Sevinç M | Brazil | 7/34 | 56(26-73) | 7/0 | 2 | -- | 2/5 | 6 |
|  |  |  |  | 34/0 | 3 | -- | 17/10 |  |
| Gonçalves PD | Brazil | 24/71 | 60(9-81) | -- | 21 | 10 | 10/14 | 7 |
|  |  |  |  | -- | 11 | 46 | 56/15 |  |
| Dall'Oglio MF | Saudi Arabia | 59/171 | 59(12-90) | 26/33 | 47 | 39 | 11/48 | 7 |
|  |  |  |  | 138/33 | 37 | 109 | 84/87 |  |
| Madbouly K | Saudi Arabia | 8/40 | 50.73(20-80) | 6/2 | 2 | 8 | 3/5 | 7 |
|  |  |  |  | 39/1 | 5 | 35 | 27/13 |  |
| Kroeger N | USA/Austria/France | 475/2121 | 61(19-97) | -- | -- | -- | -- | 7 |
|  |  |  |  | -- | -- | -- | -- |  |
| Eisenberg MS | USA | 119/984 | 65.2(36-90) | 19/100 | 108 | 119 | -- | 7 |
|  |  |  | 61.9(19-93) | 694/289 | 526 | 984 | -- |  |
| Shindo T | Japan | 14/158 | 60(23-82) | -- | \| 121 \| \| --- \| | 151 | -- | 7 |
|  |  |  |  | -- |  |  | -- |  |
| Rodriguez Faba O | USA/Spain/Italy | 725/298 | 62.1±12.1 | 9/716 | 463 | 628 | -- | 7 |
|  |  |  |  | 5/293 | 178 | 254 | -- |  |
| Bedke J | UK/Austria/Germany | 201/546 | 64.5±12.2 | 13/188 | 102 | 191 | -- | 7 |
|  |  |  | 62.4±11.9 | 438/108 | 66 | 467 | -- |  |
| Bengió RG2018 | Argentina | 40/181 | 61 | 1/39 | 26 | 37 | 10/30 | 7 |
|  |  |  | 60.5 | 138/43 | 38 | 168 | 145/36 |  |

T, MVI+; C, MVI-
